# New therapies for advanced, recurrent, and metastatic endometrial cancers

**DOI:** 10.1186/s40661-017-0056-7

**Published:** 2017-12-02

**Authors:** Vicky Makker, Angela K. Green, Robert M. Wenham, David Mutch, Brittany Davidson, David Scott Miller

**Affiliations:** 1 Gynecologic Medical Oncology Service, Department of Surgery, Memorial Sloan Kettering Cancer Center and Weill Cornell Medical College, 1275 York Avenue, New York, TX 10065 USA; 2 Department of Gynecologic Oncology, H. Lee Moffitt Cancer Center, Tampa, FL USA; 3 Division of Gynecologic Oncology, Washington University School of Medicine, St Louis, MO USA; 4 Division of Gynecologic Oncology, Duke University Medical Center, Duke Cancer Institute, Durham, NC USA; 5 Division of Gynecologic Oncology, University of Texas Southwestern Medical Center, Dallas, USA

**Keywords:** Endometrial cancer, Targeted therapy, Chemotherapy, Immunotherapy

## Abstract

Endometrial cancer is the most common gynecologic malignancy in the United States, accounting for 6% of cancers in women. In 2017, an estimated 61,380 women were diagnosed with endometrial cancer, and approximately 11,000 died from this disease. From 1987 to 2008, there was a 50% increase in the incidence of endometrial cancer, with an approximate 300% increase in the number of associated deaths. Although there are many chemotherapeutic and targeted therapy agents approved for ovarian, fallopian tube and primary peritoneal cancers, since the 1971 approval of megestrol acetate for the palliative treatment of advanced endometrial cancer, only pembrolizumab has been Food and Drug Administration (FDA)-approved for high microsatellite instability (MSI-H) or mismatch repair deficient (dMMR) endometrial cancer; this highlights the need for new therapies to treat advanced, recurrent, metastatic endometrial cancer. In this review, we discuss current and emerging treatment options for endometrial cancer, including chemotherapy, targeted therapy, and immunotherapy. The National Cancer Institute (NCI) and others are now focusing their efforts on the design of scientifically rational targeted therapy and immunotherapy trials for specific molecular phenotypes of endometrial cancer. This is essential for the advancement of cancer care for women, which is threatened by a severe enrollment decline of approximately 80% for gynecologic oncology clinical trials.

## Introduction

Endometrial cancer (EC) is the most common gynecologic malignancy in the United States, with an estimated 61,380 new cases and 11,000 deaths in 2017 [1]. The incidence of EC is increasing annually by an estimated 1–2%. The number of deaths attributed to EC are also increasing, while the mortality rate for ovarian cancer is declining [2, 3]. Obesity is a strong risk factor for the development of EC, accounting for approximately 50% of cases in Europe and the United States; it also has been associated with a relative increased risk of death of up to 6.25 [4].

Reduced birth rates, improvements in health and nutrition, and changes in the social structure of developed countries have led to an increasing elderly population, at a rate of 2.4% per year [5]. With the aging population, health promotion and disease prevention initiatives are warranted for individuals older than 50 years of age [6]. Of note, most EC diagnoses are made in women aged 45 to 74 years [3].

From 1987 to 2008, there was a 50% increase in the incidence of EC, with an approximate 300% increase in the number of associated deaths; however, no new agents were approved for treating EC. Although there are many drugs approved for the treatment of ovarian, fallopian tube, and primary peritoneal cancers, to date, there are only two FDA-approved drugs for EC, highlighting the need for new therapies to treat advanced, recurrent, metastatic EC [7].

## Review

### Types of endometrial cancer

Endometrial adenocarcinomas can be classified into two histologic categories—type 1 or type 2 [8]. Approximately 70–80% of new cases are classified as type 1 endometrial carcinomas, which are of endometrioid histology, lower grade, and often confined to the uterus at diagnosis. These tumors are estrogen-mediated, and often, women diagnosed with type 1 endometrial carcinomas are obese, with excess endogenous estrogen production. Type 1 carcinomas (estrogen dependent) have high rates of *K-ras* and PTEN loss or mutation, as well as defects in mismatch repair genes, which lead to microsatellite instability (MSI) [9–13]. Type 2 (non-estrogen dependent) carcinomas are higher-grade adenocarcinomas and are of non-endometrioid histology, occurring in older, leaner women, although an association with increasing body mass index (BMI) has been observed. Type 2 cancers have *p53* mutations, may have overexpression of human epidermal growth factor receptor 2 (HER-2/neu), and show aneuploidy [14–20]. It should be noted that there are limitations to this dualistic classification of ECs, as there is heterogeneity and often overlap of the underlying genetics; for example, many high-grade endometrioid cancers can harbor p53 mutations and behave like other type 2 cancers. A recent Gynecologic Oncology Group (GOG) study evaluated the etiologic heterogeneity of ECs and reported that women with type 2 cancers, compared with type 1 cancers, were less likely to be obese but more likely to be older, non-white, multiparous, and current smokers [21]. Women with grade 3 endometrioid carcinomas displayed characteristics that were similar to those of type 2 cancers, but more often had histories of breast cancer without tamoxifen exposure.

Uterine carcinosarcomas, a poorly differentiated subgroup of uterine carcinomas, account for less than 5% of all uterine malignancies and are rare, aggressive biphasic neoplasms that consist of high-grade malignant epithelial and mesenchymal elements [22]. Five-year progression-free survival (PFS) rates for uterine-confined disease range from 40 to 75%, compared with 20–35% for disease with extra-uterine extension [23, 24].

The Cancer Genome Atlas (TCGA) Research Network has performed the most comprehensive molecular study of EC, integrating genomic, transcriptomic, and proteomic characterizations of EC based on array and sequencing technologies in 373 primary EC surgical specimens [25]. These data revealed that EC can be classified into four molecularly phenotypically different groups: 1) DNA polymerase epsilon catalytic subunit (POLE) ultramutated (very high mutation rate, hot spot mutations in POLE, endometrioid histology, frequently grade 3 [>50%], associated with a good prognosis, comprises 1% of cases of recurrent disease, and characterized by mutations in PTEN [94%], PIK3CA [71%], PIK3R1 [65%], FBXW7 [82%], ARID1A [76%], KRAS [53%], and ARID5B [47%]); 2) MSI hypermutated (high mutation rate, microsatellite unstable, frequently with MLH-1 promoter hypermethylation, endometrioid histology, comprises approximately 25% of cases of recurrent disease, and characterized by mutations in PTEN [88%], RPL22 [33%], KRAS [35%], PIK3CA [54%], PIK3R1 [40%], and ARID1A [37%]); 3) copy-number low (lower mutation rate, microsatellite stable (MSS), endometrioid histology, grade 1/2 tumors, and characterized by mutations in PTEN [77%], CTNNB1 [52%], PIK3CA [53%], PIK3R1 [33%], and ARID1A [42%]); and 4) copy-number high serous-like (lowest mutation rate, serous, comprises approximately 25% of grade 3 endometrioid cases, poorest prognosis, and characterized by mutations in TP53 [92%], PPP2R1A [22%], PIK3CA [47%], and chromosomal instability).

The classification of EC by morphologic features is irreproducible and imperfectly reflects tumor biology. A molecular classification system based on the TCGA genomic subgroups, referred to as the Proactive Molecular Risk Classifier for Endometrial Cancer (ProMisE), was developed to confirm the feasibility and prognostic ability in a separate cohort of ECs [26]. ProMisE successfully categorized all cases and improved subgroup discrimination compared with the European Society of Medical Oncology (ESMO) risk classification system.

A TCGA analysis of 57 primary uterine carcinosarcoma tumor samples revealed extensive copy-number alterations and highly recurrent somatic mutations. Similar to endometrioid and serous endometrial carcinomas, mutations in TP53 (91%), PIK3CA (35%), PPP2R1A (28%), FBXW7 (28%), PTEN (19%), FBXW7, and KRAS (12%) were identified. A strong epithelial-to-mesenchymal transition (EMT) gene signature was observed in a subset of analyzed cases; the range of EMT scores was the largest among all tumors studied thus far by TCGA. Multiple somatic mutations and copy-number alterations in genes that are therapeutic targets were identified; 62% of tumors had one or more potentially clinically relevant mutations in the PI3K/AKT/mTOR pathway, and approximately 23% of cases had alterations in cell-cycle genes [27].

### Risk factors

Risk factors for EC include endometrial hyperplasia, unopposed estrogen therapy, tamoxifen use, obesity, reproductive factors (early menarche/late menopause, nulliparity, or polycystic ovarian syndrome), family history/genetic predisposition, and hyperinsulinemia [28–34]. Lynch syndrome, an autosomal dominant inherited cancer susceptibility syndrome, is caused by a germline mutation in mismatch repair (MMR) genes (MLH1, MSH2, MSH6, and PMS2) and accounts for 2–5% of endometrial carcinomas [35–37]. Women with Lynch syndrome have an approximate 70% lifetime risk of developing EC [38, 39]. MMR is a single-strand DNA repair mechanism critical to maintaining genomic stability. MMR genes can be lost via mutation or methylation, with MMR deficiency associated with up to 30% of all ECs [25].

Type I EC oncogenesis is primarily estrogen dependent, having a positive correlation with high circulating estrogen levels [40, 41]. Prolonged exposure to estrogens through early menarche, late menopause, or the use of hormone replacement therapy are known risk factors [42]. Furthermore, sex hormone production from adipose tissue leads to estrogen stimulation of the endometrial lining [43].

Emerging data identify hyperinsulinemia, hyperglycemia, and chronic inflammation as potentially modifiable risk factors for the development and progression of multiple malignancies, including EC. Numerous etiologies lead to the development of the metabolic syndrome; however, obesity is dominant and has rising prevalence [44].

Based on National Health and Nutrition Examination Survey (NHANES) data, since 1960, the percentages of adults classified as overweight, obese, or extremely obese have continued to increase [45]. Obese women (BMI >30 kg/m^2^), compared with normal-weight women, are at an increased risk of developing EC, with each 5 kg/m^2^ increase in BMI conferring additional risk [46, 47]. A recent meta-analysis reported that, compared with normal-weight women, the risk of developing EC was 1.34 times higher in overweight women and 2.54 times higher in obese women [48]. Outcomes for EC also are affected by obesity. A retrospective study of patients with EC managed with surgery demonstrated that obese women had significantly more perioperative complications [49]. In obese women, death rates from EC are much higher compared with death rates in obese patients with other malignancies, suggesting the importance of the angiogenic tumor microenvironment related to adiposity [46–48]. Currently, there are a myriad of studies evaluating the impact of calorie restriction and exercise in promoting weight loss in obese patients with EC (NCT02665962, NCT02665962), as well as the impact of ketogenic diet in overweight or obese patients with newly diagnosed EC (NCT03285152).

### Signaling pathways in endometrial cancer

One of the hallmarks of cancer is metabolic “addiction” to glucose, which is partially due to alterations in mitochondrial structure and function that result from genetic, epigenetic, and enzymatic alterations within cancer cells [50–53]. Current research suggests that this preferential metabolism of glucose through glycolysis may arise as a selective advantage in the hypoxic conditions experienced during early tumor development [54]. This cellular reprogramming of glucose metabolism to fuel tumor cell growth is largely thought to be driven by the AKTphosphoinositide 3-kinase (PI3K)/mammalian target of rapamycin (mTOR) pathway, which is commonly activated in endometrial carcinomas.

In fact, EC demonstrates the highest rate of PI3K pathway alterations of all solid tumors. The PI3K pathway also regulates cell growth, survival and motility, all key aspects of cancer cell biology. There are three classes of PI3K enzymes, which are grouped according to structure and function. Class IA PI3Ks are most associated with promoting carcinogenesis. Pathway activation begins with membrane-associated receptor tyrosine kinases (RTKs), such as the insulin-like growth factor receptor (IGFR), which has more than five-fold increased expression in endometrial adenocarcinoma compared with normal endometrium [55]. Upon stimulation of RTKs, PI3K phosphorylates the lipid phosphatidylinositol 4,5- biphosphate (PIP2), creating phosphatidylinositol 3,4,5- triphosphate (PIP3). PIP3 recruits protein kinase AKT to the membrane, where it is phosphorylated and activated by mTOR complex 2 (mTORC2) and 3-phosphoinositide-dependent protein kinase 1 (PDK1). Among its targets, AKT phosphorylates and inhibits tuberous sclerosis complex 2 (TSC2) within the TSC complex, which indirectly inhibits mTOR complex 1 (mTORC1). PI3K-AKT signaling activates mTORC1 [56], a key regulator of metabolism and biosynthetic processes, including activation of hypoxia-inducible factor 1 (HIF1) and other transcription factors. HIF1 stimulates glucose transporter expression on the cell surface, thereby increasing cellular glucose influx, and shifts metabolic pathways towards glycolysis through inhibitory mitochondrial pyruvate dehydrogenase kinase activation [49, 57].

Obesity, due to physical inactivity and excess caloric intake, leads to high glucose, insulin, and insulin-like growth factor 1 (IGF-1) levels. Increased signaling via the insulin/IGF-1 pathway culminates in activation of the mTOR pathway, resulting in increased cell proliferation and cancer development. Elevated glucose levels reduce 5′ adenosine monophosphate-activated protein kinase (AMPK) levels, which in turn increase mTOR stimulation and cell proliferation. Components of the mTOR pathway are often mutated, amplified, or aberrantly expressed in ECs, further supporting the link between obesity and this disease [49].

### Metastatic disease

Most women with EC are diagnosed at an early stage. The 5-year survival rate for those diagnosed with localized disease is 95%; however, women diagnosed with advanced or recurrent disease have a poor prognosis, with a 5-year survival rate of 17% [3, 58]. In a study of four GOG trials evaluating the relationship between histology and outcomes of women with advanced, recurrent EC, the median overall survival (OS) was less than 12 months, with PFS ranging from 3 to 6 months based on histology [59]. Unfortunately, most current chemotherapeutic options for advanced EC are associated with significant toxicity and limited efficacy, highlighting the need to continue with efforts to exploit the molecular underpinnings and biology of this disease for target-specific and immunotherapeutic approaches.

### Novel approaches for treating endometrial cancer

#### Adjuvant chemotherapy

Based on the findings of several GOG trials, platinum doublet chemotherapy remains the mainstay for first-line systemic therapy in patients with advanced EC. A phase 3 study of cisplatin plus doxorubicin demonstrated improved response rates and PFS (5.7 vs 3.8 months) compared with doxorubicin alone, but the regimen was not associated with increased OS (9.0 vs 9.2 months) [60]. Following this trial, the GOG-122 study accrued 396 patients with stage III or IV EC and a maximum of 2 cm of postoperative residual disease and randomized them to treatment with whole-abdominal irradiation or doxorubicin-cisplatin chemotherapy [61]. With a median 74 months of follow-up, patients in the chemotherapy arm, compared with those in the radiation arm, had a significantly improved 5-year survival rate (55% vs. 42%, respectively); however, the chemotherapy arm was also associated with greater toxicity. This pivotal study led to a paradigm shift in the management of advanced-stage EC.

To further improve efficacy, the GOG-177 study was designed to compare doxorubicin, cisplatin, paclitaxel and filgrastim support (TAP) with doxorubicin and cisplatin. Findings from the study demonstrated a significantly improved response rate (57% vs 34%, respectively), PFS (8.3 vs 5.3 months, respectively), and OS (15.3 vs 12.3 months, respectively; *p* = 0.037) with the former regimen, albeit with significantly higher patient-reported neurotoxicity [62]. In an effort to develop a less toxic regimen, GOG-209 was designed to compare carboplatin and paclitaxel (CT) to the triplet TAP regimen. This study demonstrated that CT was not inferior to TAP in terms of PFS (14 months in both arms) and OS (32 vs 38 months, respectively; HR 1.01) [63]. The toxicity profile for CT was significantly more favorable, and this regimen serves as the acceptable backbone for chemotherapy trials.

A myriad of ongoing studies using CT as the chemotherapy backbone include a phase 1 study of the selective inhibitor of nuclear export selinexor in combination with CT in patients with advanced ovarian or endometrial cancers (NCT02269293), a phase 2 study of the androgen-receptor inhibitor enzalutamide in combination with CT in advanced endometrioid EC (NCT02684227), a randomized phase 2 trial of CT compared to CT plus bevacizumab in advanced-stage or recurrent EC (NCT01770171), a phase 2 study of pembrolizumab in combination with CT in advanced EC (NCT02549209), and a randomized phase 2/3 study of CT plus metformin (NSC#91485) versus CT plus placebo as initial therapy in advanced-stage or recurrent EC (NCT02065687).

#### Chemotherapy for uterine carcinosarcomas

In the GOG-108 study, patients with advanced or recurrent carcinosarcoma treated with the combination of ifosfamide and cisplatin exhibited increased response rates and longer PFS compared with patients who received ifosfamide alone; no significant difference in survival was reported [64]. Results from a follow-up trial (GOG-161) demonstrated an increased response rate (29 vs 45%), median PFS (3.6 vs 5.8 months), and OS (8.4 vs 13.5 months) in patients receiving a 3-day regimen of ifosfamide plus paclitaxel versus paclitaxel alone [65]. As with ixabepilone for EC treatment, a nearly identical and modest response rate (12%), median PFS 1.7 months), and OS (7.7 months) were seen in 34 patients with uterine carcinosarcoma in the GOG-130F study [66]. Furthermore, as with treatment of other ECs, the use of CT in uterine carcinosarcoma is an appropriate option based on apparent equivalent efficacy and better tolerability [67].

A phase 2 trial using CT as the chemotherapy backbone and BSI-201 in advanced uterine carcinosarcomas was recently completed (NCT00687687). BSI-201, by activating gamma-H2AX, induces cell cycle arrest in the G2/M phase in tumor cell lines, and potentiates the cell cycle effects of DNA damaging modalities in tumor cell lines. Other ongoing studies include a feasibility trial of CT and galunisertib (inhibitor of the kinase domain of Type 1 TGF-B receptor) in patients with newly diagnosed or recurrent carcinosarcoma of the uterus or ovary (NCT03206177) and the phase 1 study of selinexor in combination with CT in patients with advanced ovarian or endometrial cancers (NCT02269293).

#### Advanced-disease treatments

Recent chemotherapy trials in the advanced/recurrent disease setting have not shown significant outcome improvements over prior single-agent chemotherapy studies. For example, a phase 3 randomized trial of second-line ixabepilone, an anti-tubulin epothilone, versus paclitaxel or doxorubicin in women with advanced EC failed to meet its primary objective of improving OS in the ixabepilone arm compared with the control chemotherapy arm. At interim analysis, the study of futility for OS favored the control chemotherapy arm (HR = 1.3; 95% CI: 1.0–1.7; stratified log-rank test *P* = 0.0397), and the study was discontinued based on the interim OS results [68].

#### Hormonal strategies and antibody drug conjugates

Given the endocrine sex hormone relationship with most ECs, agents that target these receptors and pathways have been evaluated and are in clinical use for EC of low-grade endometrioid histology; however, they are associated with limited efficacy. Megestrol acetate, a progestin, was approved more than 40 years ago for the palliative treatment of recurrent, metastatic breast and endometrial cancers. More recently, the GOG-153 study evaluated the combined hormonal strategy of alternating tamoxifen and megestrol acetate based on the hypothesis that tamoxifen increases the expression of progesterone receptors and thereby increases the efficacy of megestrol acetate. Megestrol acetate at 80 mg twice daily every 3 weeks, alternating with tamoxifen 20 mg twice daily every 3 weeks, was associated with an overall response rate of 27% [69]. Responses were attenuated by grade, which is correlated with hormone receptor status, with rates of 38%, 24%, and 22% for grade 1, 2, and 3 disease, respectively. Estrogen reduction by aromatase inhibitors, specifically anastrozole and letrozole, has shown little activity in EC in two prior studies [64, 65].

Elevated cyclin-dependant kinase 4 (CDK4) expression is observed in 34% to 77% of endometrioid endometrial cancers (EECs) and is considered to be an early event of neoplastic transformation in EEC [70]. CDK4/6 mediate the transition from G1 to S phase by associating with D-type cyclins and regulating the phosphorylation state of retinoblastoma. CDK4/6SA is significantly higher (*P* = 0.002) in pathologically low-risk patients (not receiving adjuvant chemotherapy, *n* = 74) than in intermediate- or high-risk patients (receiving adjuvant chemotherapy, *n* = 35). Patients with high CDK4/6SA (>3.0) have significantly (*P* = 0.024) shorter PFS than those with low CDK4/6SA (<3.0) [71]. CDK4/6 inhibitors restore cell-cycle control and halt tumor growth. In an effort to improve the efficacy of treatment in this setting, a randomized phase 2 trial of palbociclib (CDK4/6 inhibitor) in combination with letrozole versus placebo in combination with letrozole for patients with estrogen receptor (ER)-positive advanced or recurrent EC (NCT02730429) and a phase 2 trial of ribociclib (cyclin D1 and CDK4/6 inhibitor) and letrozole in ER-positive advanced ovarian, fallopian tube, primary peritoneal carcinomas and EC (NCT02657928) are currently recruiting patients.

Luteinizing hormone-releasing hormone receptors (LHRH-Rs) mediate antiproliferative activity in endometrial cell lines, and approximately 80% of ECs express LHRH-Rs, offering a potentially useful target in these tumors [66, 67]. Zoptarelin doxorubicin is an [D-Lys6]LHRH linked to doxorubicin, with activity in LHRH-R-positive cancer cell lines [72, 73]. Zoptarelin doxorubicin is internalized via LHRH-R and induces apoptosis without activating the MDR-1 efflux pump system, and it is less toxic than doxorubicin [73–76]. In an initial study of 17 women with ovarian, endometrial, or breast cancer who received various doses of zoptarelin doxorubicin, 3 patients who received 160 mg/m^2^ and 3 patients who received 267 mg/m^2^ (maximally tolerated dose) responded [77]. In a phase 2 study of 43 patients with LHRH-R-positive advanced EC, 2 patients achieved complete remission and 8 achieved partial remission following zoptarelin doxorubicin administration [78]. The overall objective response and stable disease rates were 23% and 47%, respectively. The ZoptEC phase 3 trial compared the efficacy and safety of zoptarelin doxorubicin to doxorubicin alone. Patients were centrally randomized in a 1:1 ratio and received either zoptarelin doxorubicin (267 mg/m^2^) or doxorubicin (60 mg/m^2^) intravenously every 3 weeks for up to 9 cycles. The median OS period for patients treated with zoptarelin doxorubicin was 10.9 months compared with 10.8 months for patients treated with doxorubicin. This was not a statistically significant, clinically meaningful increase in OS, and thus the ZoptEC phase 3 clinical study did not meet its primary endpoint (unpublished data).

Folate receptor alpha (FRα) expression is associated with high-grade, advanced-stage EC and a poor prognosis, particularly in serous-type tumors, [79, 80], thus providing an attractive candidate for novel, targeted therapeutic strategies in advanced EC. Mirvetuximab soravtansine is an antibody-drug conjugate (ADC) comprised of an FRα-binding antibody, cleavable linker, and the maytansinoid DM4, a potent tubulin-targeting agent. A phase 1 expansion study of mirvetuximab soravtansine in patients with EC is currently ongoing (NCT02606305).

### Other targeted therapies

#### Antiangiogenic therapies

Vascular endothelial growth factor (VEGF) is expressed in most ECs and is associated with higher histologic grade, lymphovascular space invasion, lymph node metastasis, and deep myometrial invasion [81–86]. In the GOG-229E study, bevacizumab therapy led to an overall response rate of 14% in 52 women with persistent or recurrent EC treated with one or two prior cytotoxic regimens [87]. Multiple phase 2 trials have demonstrated improved PFS with bevacizumab monotherapy or in combination with an mTOR inhibitor [88, 89]. The ongoing GOG-86P trial of bevacizumab with cytotoxic agents has demonstrated a potential survival benefit [90], and final results are anticipated in the near future.

Other anti-angiogenic agents have been investigated but have shown limited activity; these agents include thalidomide, aflibercept, sorafenib, and the small-molecule tyrosine kinase inhibitors (TKIs) dovitinib, nintedanib, brivanib, and sunitinib [91–94]. The GOG-229F trial of aflibercept (VEGF ligand binding fusion) in 44 patients with advanced EC met its study endpoint of PFS at 6 months but was associated with significant toxicities at the studied dose and schedule [95]. Cediranib, a multi-target TKI, targets VEGF 1–3 and platelet-derived growth factor β (PDGFβ) receptors, as well as c-Kit. The recent GOG-229 J study of cediranib in advanced EC demonstrated its sufficient activity and tolerability as monotherapy treatment (Table 1) [87, 92, 95–101].

**Table 1:** Anti-angiogenic Therapies

Study Drug	Target	Prior Lines of Therapy	Patients	ORR	mTTP/PFS (months)	mOS (months)
Dalantercept [98]	BMP9/10	1–2	28	0%	2.1	14.5
Trebananib [99]	Tie2 Receptor	1–2	32	3.1%	2	6.6
Cediranib [96]	VEGF/c-kit	1–2	48	12.5%	3.7	12.5
Sunitinib [91]	VEGF/KIT/PDGFR	≤ 1	33	18.2%	3.0	19.4
Nintedanib [92]	VEGF/FGFR/PDGFR	1–2	32	9.4%	3.1	10.1
Lenvatinib [97]	VEGFR/FGFR/RET/KIT/PDGFRβ	1–2	133	14.3%	5.6	10.6
Aflibercept [95]	VEGFR	1–2	44	7%	2.9	14.6
Bevacizumab [87]	VEGFR	1–2	52	13.5%	4.2	10.6
Sorafenib [100]	VEGF/Raf/Ras	≤ 1	39	5%	3.2	11.4
Thalidomide [101]	VEGFR/bFGF	1–2	21	12.5%	1.7	6.3

*ORR* objective response rate, *mTTP* median time to progression, *PFS* progression-free survival, *mOS* median overall survival

Lenvatinib, an oral receptor TKI, targets VEGF receptors 1–3, fibroblast growth factor receptos1–4, RET, KIT, and PDGFβ. Confirmed complete responses and partial responses were observed in 19 patients (14%) and 29 patients (22%) treated with lenvatinib by independent review and investigator assessment, respectively [97]. The median PFS was 5.4 months, and the median OS was 10.6 months. Lenvatinib is being developed further in combination with immunotherapy.

#### EGFR pathway inhibitors

In EC, epidermal growth factor receptor (EGFR) overexpression is common, and is associated with deep myometrial invasion, tumor grade, and a poor prognosis [102–104]. Low response rates have been reported for the oral EGFR inhibitors gefitinib and erlotinib in phase 2 trials [105, 106].

HER2/neu is a member of the human epidermal growth factor receptor (HER/EGFR/ERBB) family. HER2/neu overexpression leads to alterations in cell proliferation, migration, differentiation, and survival, as well as the upregulation of the Ras/Raf/MAPK and PI3K/AKT/mTOR pathways [107]. HER2/neu overexpression is seen in advanced type 2 cancers and is associated with a poor prognosis [108, 109]. ERBB2/HER2 is an RTK that mediates signaling via the PI3K and mitogen-activated protein kinase (MAPK) pathways. Importantly, ERBB2 was focally amplified with protein overexpression in 25% of the serous or serous-like tumors based on TCGA data [25]. A phase 2 trial of the HER2/EGFR inhibitor lapatinib in molecularly unselected advanced EC revealed limited activity, with an ORR of 3.3% and median PFS of 1.8 months [110]. A randomized phase 2 trial (NCT01367002) evaluating CT in combination with trastuzumab, which as a single agent showed limited activity in a previous phase 2 trial (ORR, 0%; median PFS, 1.8 months) [111], closed due to poor accrual. Ongoing trials of ado-trastuzumab emtansine and afatinib (irreversible EGFR, HER2, and HER4 inhibitor) for patients with EC and HER2-amplified or mutant cancers are accruing (NCT02675829, NCT02491099).

#### Inhibitors of the PI3K/Akt/mTOR pathway

Since the initial studies of nearly 20 years ago, a myriad of approaches to target this pathway have been explored. The initial studies of rapamycin analogs (rapalogs) that bind directly to and allosterically inhibit mTOR1 revealed modest but reproducible antitumor activity across the serous, endometrioid and clear cell histologic subtypes, with some patients experiencing prolonged stable disease. Several phase 2 trials have investigated the use of mTOR inhibitors as single agents in advanced EC. The objective response rates (ORRs) in those studies ranged form 0% to 24%, and responses were higher in chemotherapy-naïve patients (Table 2) [112–117].

**Table 2:** Single-Agent mTOR Inhibitor Studies in Endometrial Cancer

Agent	Patients	Prior Chemotherapy Regimens	Molecular Selection of Patients	Objective Response Rate	Other Activity
Temsirolimus [112]	29	None	No	24%	SD ≥ 8 weeks: 69%
	25	1–2	No	4%	SD ≥ 8 weeks: 46%
Everolimus [113]	28	1–2	No	0%	SD: 43%
Everolimus [114]	44	1–2	No	9%	SD: 27%
Ridaforolimus IV [115]	45	1–2	No	11%	CBR: 29%
Ridaforolimus PO [116]	30	Adjuvant only	No	9%	SD: 52.9%
Ridaforolimus PO [117]	64	1–2	No	0%	SD: 35%

*IV* intravenous, *PO* oral, *SD* stable disease, *CBR* clinical benefit rate

The modest activity shown in these studies could be secondary to the existence of intra- or inter-pathway feedback loops (e.g., MAPK pathway) and from the incomplete blockage of the pathway provided by the rapalogs. Correlative analyses of archival biospecimens have failed to identify a predictive biomarker in EC, and these initial studies also did not enrich for patients with abnormalities in the PI3K/Akt/mTOR pathway. These studies may have been strengthened by limiting eligibility to patients with alterations in this pathway, although it is also possible that a single or multiple target biomarkers may be insufficient to predict for response due to the inherent complexity of this pathway.

Rapalogs have also been evaluated in combination trials. A phase 2 study of everolimus plus letrozole reported a response rate of 32% and a clinical benefit rate (CBR) of 40% [118]. Interim results of an ongoing phase 2 trial of everolimus, letrozole and metformin showed a partial response rate of 29%, and 38% of patients achieved stable disease [119]. The GOG-248 study of temsirolimus with or without megestrol acetate in 71 patients revealed that adding the combination of megestrol acetate and tamoxifen to temsirolimus did not enhance activity. The study was closed early due to an excess of venous thrombosis in the combination arm [120].

Due to the incomplete inhibition of mTORC1 targets by rapalogs, and the feedback loops that exist, activation of upstream PI3K signals [54, 55] can result. It has been hypothesized that newer PI3K pathway agents, which target further upstream in the pathway, will be more clinically effective. Numerous phase 1b/2 clinical trials evaluating catalytic mTOR, AKT, pan-PI3K, and dual PI3K/mTOR inhibitors are underway.

mTOR inhibitors have also been combined with chemotherapy. Two phase 1 trials in solid tumors using CT with either ridaforolimus [121] or temsirolimus [122] showed response rates of 25% and 82%, respectively, in EC populations. However, a randomized phase 2 trial (GOG-86P) comparing CT with either temsirolimus or bevacizumab or carboplatin plus ixabepilone and bevacizumab to CT showed improved OS when bevacizumab, but not temsirolimus, was added to CT [90].

#### Non-rapalog PI3K/AKT/mTOR inhibitors

There are multiple completed or ongoing single-agent phase 2 clinical trials examining non-rapalog PI3K/mTOR agents in EC. A phase 2, two-stage, two-arm PIK3CA mutation stratified trial of MK-2206, an allosteric inhibitor of AKT, of previously treated endometrial cancer also revealed limited single-agent activity in both mutant (1 partial response) and wild-type (1 partial response) EC populations, although activity was detected in serous histology tumors with exploratory analysis, revealing that all patients with a 6-month PFS had serous EC. This study may have suffered from small patient numbers in the mutant group, as well as poor drug tolerance [123]. A phase 2 trial of the pan class I PI3K inhibitor pilaralisib demonstrated minimal activity, with an ORR of 6%, as did the phase 2 MAGGIE study of GDC-0980, a dual PI3K/mTOR inhibitor, which also demonstrated an ORR of 6% and limited antitumor activity [124, 125]. Both studies were limited in that they did not require an alteration in the PI3K/Akt/mTOR axis. Similarly, a phase 2 double-strata (low grade vs high grade) trial of BKM120, a pure PI3K inhibitor, in previously treated EC demonstrated an ORR of 0%, and was discontinued early due to excessive toxicity [126]. A phase 2 trial of LY3023414, a PI3K/mTOR inhibitor (NCT02549989), in EC with PI3K pathway activation without concurrent resistance mutations is ongoing. HER2/neu gene amplification and PIK3CA driver mutations are common in uterine serous carcinoma. Preclinical studies have shown that HER2-amplified serous cell lines were more sensitive to growth inhibition by PI3K inhibitors than HER2 non-amplified serous EC cell lines, a potential future direction [127]. Of additional interest is the combination with poly (ADP-ribose) polymerase (PARP) inhibitors, as drugs targeting the PIK3/AKT/mTOR pathway may interfere with DNA repair mechanisms, as described below.

#### Metformin

Metformin is an oral biguanide agent that is known to inhibit cellular proliferation and induce apoptosis, potentially through inhibition of Mitochondrial complex 1 and AMPK activation and mTOR inhibition [128–131]. An association between metformin use and improved outcomes in patients on prior single-agent mTOR inhibitors, as well as in the everolimus and letrozole combination, has been shown [118, 132]. Currently, there are numerous metformin chemoprevention studies, as well as studies of metformin combinations with standard chemotherapy (NCT02065687) and with hormonal and mTOR agents (NCT01797523).

#### PARP inhibitors

Preclinical studies have shown that inhibition of the PI3K/akt/mTOR pathway may sensitize EC cell lines to PARP inhibitors, and that loss of PTEN function may predict sensitivity to PARP due to a synthetic lethality process. This appears to be particularly true in a low-estrogenic setting [123, 133]. A phase 2 study of the PARP inhibitor niraparib in recurrent EC (NCT03016338) is active but not yet recruiting.

In addition, a phase I study is exploring the role of the PARP inhibitor olaparib in combination with the mTORC1/2 inhibitor AZD2014 or the AKT inhibitor AZD5363 for gynecological cancers, including advanced ECs (NCT02208375).

#### Immunotherapy

Immune checkpoint inhibitors in the treatment of EC, although potentially promising, have had until recently limited reportable data. Programmed cell death-1 (PD-1) and its ligand PD-L1 are expressed on the tumor-infiltrating immune cells of 61% to 80% of primary ECs [134, 135] and in 100% of metastatic ECs [134, 135]. Presence of tumor-infiltrating lymphocytes is also an independent prognostic factor in type I and II ECs [136]. The high mutation load in the POLE-mutated and MSI-H EC subgroups is correlated with PD-1 expression [133]. Approximately 26% of recurrent ECs harbor mismatch repair deficiency (MMD-D) or POLE-E exomuclease domain mutations (POLE EDM) in the recurrent disease setting, and may be excellent candidates for PD-1 targeting immunotherapies [137]. The vast majority of recurrent ECs are the copy-number low endometrioid and copy-number high serous-like ECs, which may warrant more tailored immunotherapy and combination treatment approaches.

A phase 2 study evaluating the clinical activity of the PD-1 inhibitor pembrolizumab in patients with colorectal cancer demonstrated an immune-related PFS rates of 78% in patients with MMR-deficient cancer and 11% in patients with MMR-proficient cancer, demonstrating that MMR status predicts the clinical benefit of pembrolizumab [138]. In preliminary results from the phase 1b KEYNOTE-028 study, there was a partial response of 13% among 24 pretreated patients with advanced EC and PD-L1 expression ≥1% [139].

Pembrolizumab recently was granted FDA accelerated approval for tissue or site agnostic use in the treatment of patients with unresectable or metastatic solid tumors, including EC, associated with MSI-H or MMR-deficient disease. This was the FDA’s first tissue or site agnostic approval, which was based on data from five single-arm, multi-cohort, multi-center, clinical trials of 149 patients with MSI-H or MMR-deficient disease. The overall ORR based on independent review was 39.6%, with 11 complete responses and 48 partial responses. Responses lasted 6 months or longer in 78% of patients who responded.

Observations in several mouse models have shown that the oral TKI lenvatinib appears to significantly decrease the tumor-associated macrophage population, leading to increased antitumor activity and upregulation of PD-1 signal inhibitors [140, 141]. A phase 1b/2 trial of lenvatinib plus pembrolizumab in patients with selected solid tumors, including EC, is ongoing (NCT02501096). Interim results in 23 patients with advanced EC revealed an ORR of 52% by independent review. Importantly, responses were seen in both MSI-high and MSS patients [142].

Multiple monotherapy trials (NCT02628067, NCT02912572, NCT02899793, NCT02630823), combination immunotherapy trials (NCT03015129 NCT02982486), and immunotherapy trials in combination with paclitaxel and carboplatin (NCT02549209) are planned or ongoing.

## Conclusions

Based on the increasing incidence and mortality associated with EC, and with only one recently approved therapy for a subset of patients with advanced EC, the NCI has published priorities for research on treating EC, with a goal to integrate molecular and/or histologic stratification into EC management. A recent international survey reported that 94% of participants supported the concept of treating patients in appropriate clinical trials. Since 2011, there has been a severe enrollment decline of 80% for gynecologic oncology clinical trials due to decreased federal funding and the consolidation of cooperative groups within the NCI. A focus on designing clinical trials to study new rationally combined therapeutic agents, including molecularly targeted agents and immunotherapy, in patients with EC is essential to the advancement of cancer care and the improvement of outcomes among women with advanced EC.
